# Clinical significance of MMP-9 overexpression in endometrial cancer: A PRISMA-compliant meta-analysis

**DOI:** 10.3389/fonc.2022.925424

**Published:** 2022-10-26

**Authors:** Xia Li, Li Zha, Bo Li, Rong Sun, Jianhua Liu, Hongwei Zeng

**Affiliations:** ^1^ Department of Critical Medicine, Chengdu Women’s and Children’s Central Hospital, School of Medicine, University of Electronic Science and Technology of China, Chengdu, China; ^2^ Department of Gynaecology and Obstetrics, Chengdu Women’s and Children’s Central Hospital, School of Medicine, University of Electronic Science and Technology of China, Chengdu, China

**Keywords:** MMP-9, endometrial cancer, clinical progression, prognosis, meta-analysis

## Abstract

**Objective:**

Several studies have found that MMP-9, one of the extracellular matrix-degrading proteinases, was involved in EC’s (endometrial cancer) clinical progression and prognosis. However, the results involving the associations of MMP-9 expression with risk, clinical features and prognosis of EC were conflicting. Therefore, we performed a systematic review and meta-analysis to clarify the correlation of MMP-9 expression with EC.

**Methods:**

Relative studies involving the associations between MMP-9 expression and EC were retrieved from PubMed, Embase, Web of Science and CNKI (China National Knowledge Infrastructure) electronic databases. OR (odds ratio) with 95% CI (confidence interval) was applied to evaluate the associations of MMP-9 expression with risk and clinical features of EC. Furthermore, we evaluated the role of MMP-9 expression in prognosis of EC using HR and 95% CI. The funnel plots and Begg test were used to assess the publication bias.

**Results:**

A total of 28 eligible studies were acquired from Pubmed, Embase, Web of science and CNKI databases. We found MMP-9 overexpression was significantly associated with the risk of EC (OR = 11.02, 95% CI = 7.51-16.16, *P* < 0.05). In the meantime, MMP-9 overexpression was significantly associated with the tumor grade, FIGO stage, lymph node metastasis and myometrial invasion (Tumor grade: OR = 1.68, 95% CI = 1.09-2.58, *P* < 0.05; FIGO stage: OR = 3.25, 95% CI = 1.73-6.08, *P* < 0.05; Lymph node metastasis: OR = 2.98, 95% CI = 1.27-7.03, *P* < 0.05; Myometrial invasion: OR = 2.42, 95% CI = 1.42-4.12, *P* < 0.05) in Asians. In addition, the overall results showed that MMP-9 overexpression predicted a worse prognosis of EC (OR = 1.82, 95% CI = 1.01-2.62, *P* < 0.05).

**Conclusions:**

MMP-9 overexpression might be a potential predictor of poor clinical progression and prognosis of EC.

## Introduction

EC is the most common gynecologic malignancy in developed countries, while its incidence and mortality are rising (1). Most of the patients were diagnosed at 70 years or older (2). In the past several years, estrogen therapy, tamoxifen therapy, and surgical treatment significantly improved the survival rates of EC patients. However, 42,000 women still died of EC (3). Early menstruating, late menopause, infertility, polycystic ovary syndrome, increased age, hypertension and diabetes increased the risk of EC. It has been reported that obesity and conditions associated with metabolic syndrome were significantly linked with the development of EC. Obesity rates continued to rise in developed countries, which might aggravate the occurrence of EC (4). In addition, racial disparity in death rates of EC patients was found in genetic studies (4). These studies suggested that many risk factors increased EC mortality. Although the 5-year survival of EC patients with early stage was estimated to be 90%, those patients with advanced stage had a worse prognosis of EC (5). Therefore, identification of novel and reliable markers for the diagnosis, prediction for clinical progression and prognosis of EC were urgently needed.

Endometrial carcinoma could invade the basement membrane and myometrium through gelatinase, penetrating the lymphatic vascular lumen and spreading (6). MMP-9 gene was located at chromosome 20q13.12 which encoded Gelatinase B. Gelatinase B could degrade gelatin, collagen and elastin through proteolytic cleavage to regulate extracellular matrix (ECM) remodeling (7). Furthermore, Gelatinase B could directly cleave polypeptides after MMP-9 was secreted into the extracellular space (8). Therefore, MMP-9 was involved in many biological processes such as proteolytic degradation of ECM, cleavage of cell surface proteins and alteration of cell-cell or cell-ECM interactions (9). Published studies showed that MMP-9 significantly affected tumor invasion, metastasis, angiogenesis and tumor microenvironment (9–37). Therefore, MMP-9 might be a potential biological target for prediction and treatment of EC. However, the expression of MMP-9 in EC patients at different stages was still controversial in published studies (10–37). Therefore, the meta-analysis carried out a quantitative analysis to explore whether the high MMP-9 expression predicted EC’s risk, clinical progression and prognosis.

## Methods

### Search strategy

The meta-analysis was conducted according to the PRISMA 2015 statement (38). The search strategy of “(“Matrix Metalloproteinase 9”[Mesh]) AND “Endometrial Neoplasms”[Mesh]” were used to searched all studies involving the associations of MMP-9 expression with risk, clinical outcome and prognosis of EC from PubMed, Embase, Web of Science, and CNKI databases until April 2022. The following search terms were also used: “MMP-9”, “matrix metallopeptidase 9”, “prognosis”, “survival”, “neoplasms”, “EC”, “endometrial carcinoma” and “carcinoma of endometrium”. In addition, references in the eligible literature were reviewed to obtain the relevant articles.

### Study selection criteria

The literature’s inclusion and exclusion criteria were established to select and eliminate retrieved literature. All included articles should meet the inclusion criteria: 1) Studies evaluating the role of MMP-9 in the risk, clinical progression, and prognosis of EC; 2) Articles providing enough data to calculate the ORs and 95% CI; 3) Literature containing HRs with 95% CI or survival curve about the prognosis of EC. 4) Studies that the detection method of MMP-9 protein expression was IHC. Studies were excluded if they met the following exclusion criteria: 1) studies with insufficient data for calculating the OR, HR and 95% CI; 2) publications with duplicate data; 3) studies carried out in cells or animals. Two authors independently identified the eligible studies according to the inclusion and exclusion criteria.

### Data extraction

Two reviewers independently extracted relevant data from the included studies. The following information was extracted: first author’s name, year of publication, country, ethnicity, disease type, time of follow-up, the detection method of MMP-9 protein expression, cut-off values of MMP-9 protein expression and HRs with 95% CI about overall survival time of EC. If studies only provided a survival curve about the overall survival time of EC patients, we used Engauge Digitizer 4.1 software (http://digitizer.sourceforge.net/) to extract the HRs and 95% CI (39). The quality of included studies was assessed with the Newcastle Ottawa Scale (NOS) table (40). The scores of eligible studies were from 0 to 9, while 7 to 9 scores were considered as high-quality.

### Statistical analysis

Chi-squared test and *I* ^2^ statistic were applied to assess the heterogeneity among studies, and *I* ^2^ > 50 or *P*-value < 0.05 presented significant heterogeneity (41, 42). The random-effects model was adopted if significant heterogeneity existed, and the fixed-effects model was used when heterogeneity was not found (43). We drew the funnel plots by conducting the Begg’s test to evaluate the publication bias (44). Sensitivity analysis was performed to test the stability of the pooled results. In addition, subgroup analysis was used to examine the source of heterogeneity among the included studies. All statistical analysis of the present study were performed with STATA 12.0 software (Stata Corp., College Station, TX, USA). All statistical tests were two-sided, and a *P*-value < 0.05 was considered statistical significance.

## Results

### Study inclusion and characteristics

**Figure 1:**
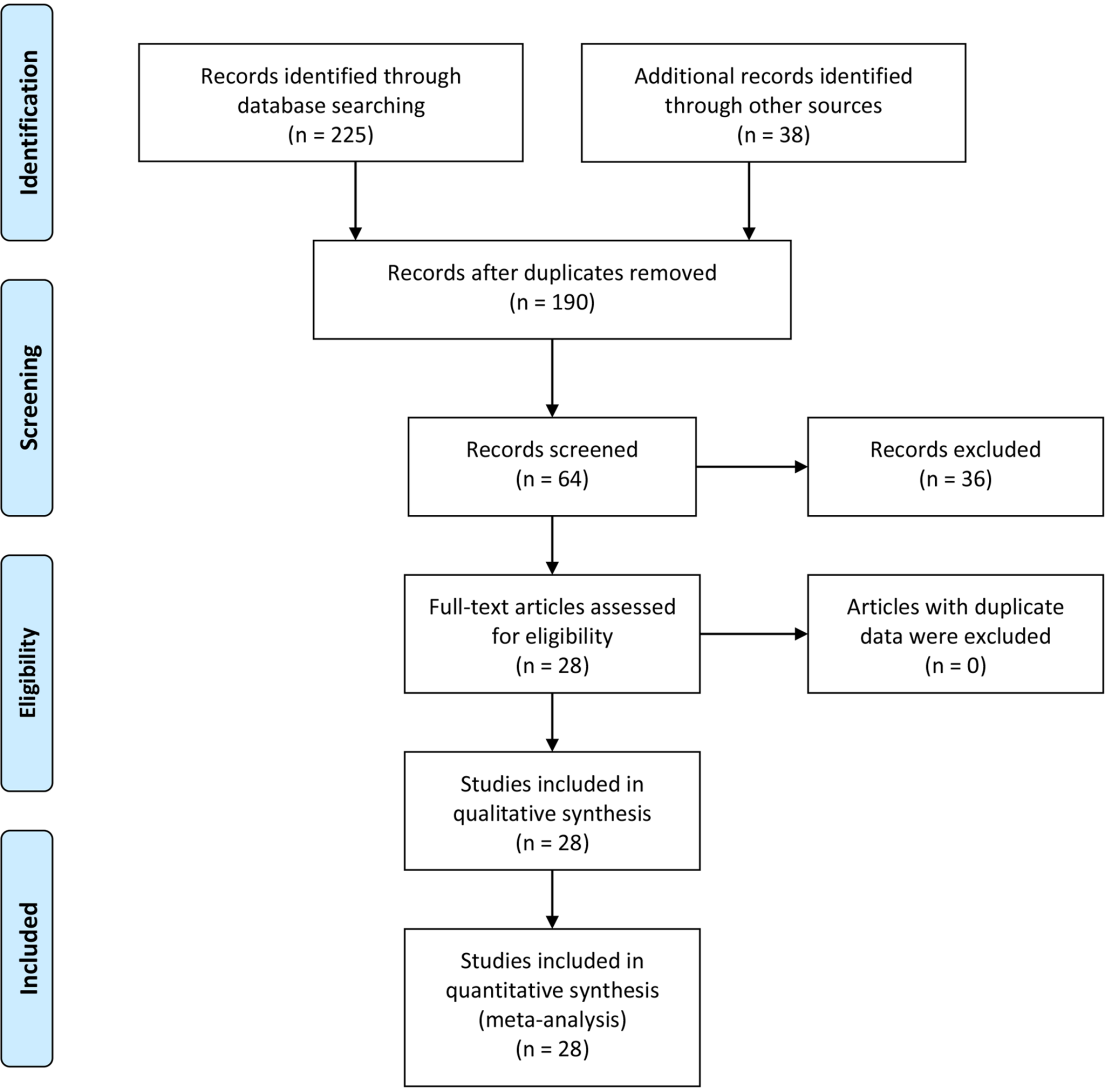
Flow chart of eligible studies selection process.

The initial search based on the inclusion and exclusion criteria identified 190 articles from electronic databases. Among them, 70 reports were duplicated and therefore were removed. Moreover, three articles were review types, so they were excluded. After titles and abstracts were read, 23 studies were excluded since they were unrelated to the associations of MMP-9 expression and risk, clinical features or prognosis of EC. In addition, 13 articles were excluded because of insufficient data. Finally, 28 eligible studies were included in the meta-analysis (10–37) (**Figure 1** and **Table 1**). In the included studies, 8 reports were carried out in Caucasians and 20 articles were performed in Asians. In addition, the information of included studies for the analysis of associations between MMP-9 expression and EC clinical features was presented in **Supplementary Table 1**.

**Table 1:** Study characteristics of the included studies for the risk and prognosis of EC.

Author	Reference	Time	Country	Ethnicity	Method	Histology	Number of NT	Number of CT	NT		CT		NOS	Cut-off
									MMP9-	MMP9+	MMP9-	MMP9+	
Ma	17	2020	China	Asians	IHC	EC	18	49	15	3	14	35	7	5%
Assaf	18	2018	Egypt	Mixed	IHC	EC	10	25	10	0	2	23	7	None
Gan	19	2018	China	Asians	IHC	EC	100	100	96	4	32	68	7	0%
Miao	20	2017	China	Asians	IHC	EC	60	60	49	11	13	47	8	30%
He	21	2016	China	Asians	IHC	EC	20	39	12	8	11	28	8	5%
Wu	22	2016	China	Asians	IHC	EC	45	60	37	8	14	46	7	25%
Wang	23	2015	China	Asians	IHC	EC	40	70	24	16	25	45	7	0%
Zhang	24	2013	China	Asians	IHC	EC	15	56	12	3	22	34	7	5%
Zhang	25	2013	China	Asians	IHC	EC	10	37	8	2	9	28	7	10%
Gao	26	2013	China	Asians	IHC	EC	27	73	20	7	12	61	7	0%
Yu	27	2012	China	Asians	IHC	EC	30	128	25	5	74	54	7	0%
Wang	28	2012	China	Asians	IHC	EC	20	43	17	3	10	33	7	5%
Liu	29	2012	China	Asians	IHC	EC	26	42	19	7	13	29	7	5%
Lu	30	2011	China	Asians	IHC	EC	18	60	15	3	13	47	7	5%
Wu	31	2011	China	Asians	IHC	EC	27	73	20	7	12	61	7	0%
Meng	32	2010	China	Asians	IHC	EC	80	180	57	23	39	141	7	10%
Gao	33	2009	China	Asians	IHC	EC	22	74	20	2	24	50	7	0%
Zhang	34	2008	China	Asians	IHC	EC	10	70	8	2	16	54	7	0%
Zhang	35	2006	China	Asians	IHC	EC	12	40	6	6	2	38	7	0%
									Num.	Survival analysis	Source of HR	HR	95% CI	
Yu	27	2012	China	Asians	IHC	EC	108	1.68	108	OS	Survival cure	1.68	0.77-3.71	0%
Aglund	41	2004	Finland	Caucasians	IHC	EC	90	2.97	90	OS	Survival cure	2.97	1.01-8.70	50%
Liu	37	2014	China	Asians	IHC	EC	120	1.8	120	OS	Survival cure	1.8	1.06-3.06	0%

IHC, immunohistochemistry; NOS, Newcastle Ottawa Scale; OS, overall survival; Num, number; HR, hazard ratio; EC, endometrial cancer; NT, normal tissue; CT, cancer tissue; EC, endometrial cancer.

### Meta-analysis results

**Figure 2:**
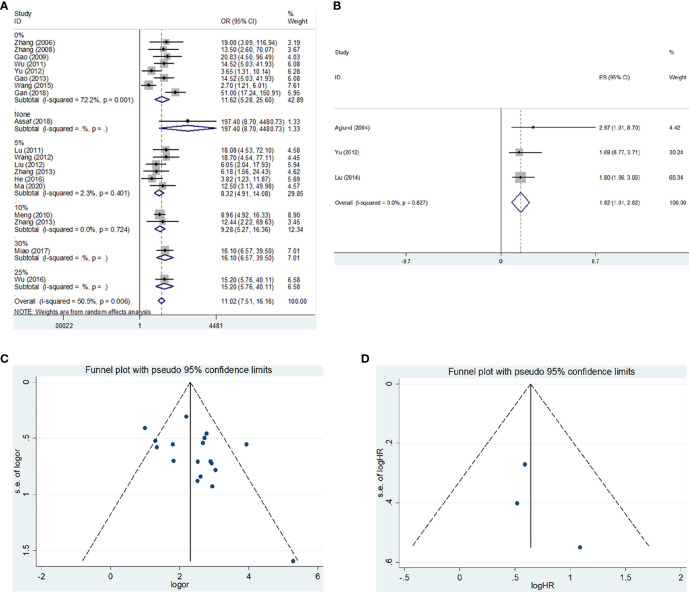
The forest plot and funnel plots for the correlations of MMP-9 overexpression with risk and prognosis of EC. **(A)** Forest plot for the risk of EC. **(B)** Forest plot for the overall survival of EC. **(C)** Funnel plot for the risk of EC. **(D)** Funnel plot for the overall survival of EC. OR, odds ratio; HR, hazard ratio; EC, endometrial cancer.

The pooled results revealed that there was a significant association between MMP-9 overexpression and risk of EC (OR = 11.02, 95% CI = 7.51 – 16.16, *P* < 0.05). Small heterogeneity was observed (*I* ^2^ = 50.5, *P* = 0.006), and a random-effects model was applied. Moreover, subgroup analysis based on ethnicity or cut-off values was performed. The results showed that MMP-9 expression was significantly correlated with risk of EC in Asians (OR = 10.55, 95% CI = 7.27 – 15.30, *P* < 0.05). In the meantime, the subgroup analysis based on cut-off values indicated that high expression of MMP-9 was still an increased risk for EC (cut-off value: 0%, OR = 11.62, 95% CI = 5.28 – 25.60, *P* < 0.05; 5%, OR = 8.32, 95% CI = 4.91 – 14.08, *P* < 0.05; 10%, OR = 9.28, 95% CI = 5.27 – 16.36, *P* < 0.05). Moreover, heterogeneity among the included studies significantly decreased in the subgroup analysis. Thus, ethnicity and cut-off values of MMP-9 expression might contribute to the heterogeneity (**Figure 2** and **Table 2**).

**Table 2:** Meta-analysis of the correlations of MMP-9 expression with risk, clinical features and prognosis of EC.

Characteristics	Studies	Forest plot analysis		Heterogeneity analysis		Begg test
		OR (95% CI)	*P*	*I* ^2^ (%)	*P*	*P*
Risk (Normal vs EC)	19	11.02 (7.51-16.16)	< 0.05	50.50	0.006	0.234
Caucasian	1	197.0 (8.70-4480.73)	< 0.05	–	–	–
Asian	18	10.55 (7.27-15.30)	< 0.05	48.10	0.012	0.544
Tumor grade (G1 vs G2+G3)	21	1.55 (1.12-2.15)	< 0.05	42.90	0.020	0.027
Caucasian	4	1.33 (0.88-2.01)	> 0.05	0.00	0.911	0.497
Asian	17	1.68 (1.09-2.58)	< 0.05	53.60	0.005	0.127
Tumor stage (I vs II+III)	14	2.30 (1.35-3.92)	< 0.05	40.90	0.055	0.033
Caucasian	4	1.03 (0.61-1.74)	> 0.05	0.00	0.485	0.042
Asian	10	3.25 (1.73-6.08)	< 0.05	24.10	0.222	0.089
Lymph node metastasis (No vs Yes)	19	2.66 (1.20-5.90)	< 0.05	77.60	0.000	0.726
Caucasian	2	0.88 (0.13-5.86)	> 0.05	35.10	0.214	–
Asian	17	2.98 (1.27-7.03)	< 0.05	79.10	0.000	0.711
Myometrial invasion (< 1/2 vs > 1/2)	14	2.20 (1.36-3.57)	< 0.05	55.00	0.007	0.298
Caucasian	2	1.19 (0.40-3.56)	> 0.05	17.50	0.271	–
Asian	12	2.42 (1.42-4.12)	< 0.05	57.50	0.007	0.217
Vascular invasion (No vs Yes)	4	1.76 (0.61-5.07)	> 0.05	56.70	0.074	1.000
Caucasian	2	1.05 (0.09-11.79)	> 0.05	58.90	0.119	–
Asian	2	2.67 (1.27-5.60)	< 0.05	0.00	0.617	–
Menopausal status (Premenopause vs Postmenopause)	4	1.14 (0.77-1.68)	> 0.05	45.80	0.137	0.497
Survival	3	1.82 (1.01-2.62)	< 0.05	0.00	0.827	0.602

OR, odds ratio; CI, confidence interval; EC, endometrial cancer; EC, endometrial cancer.

**Figure 3:**
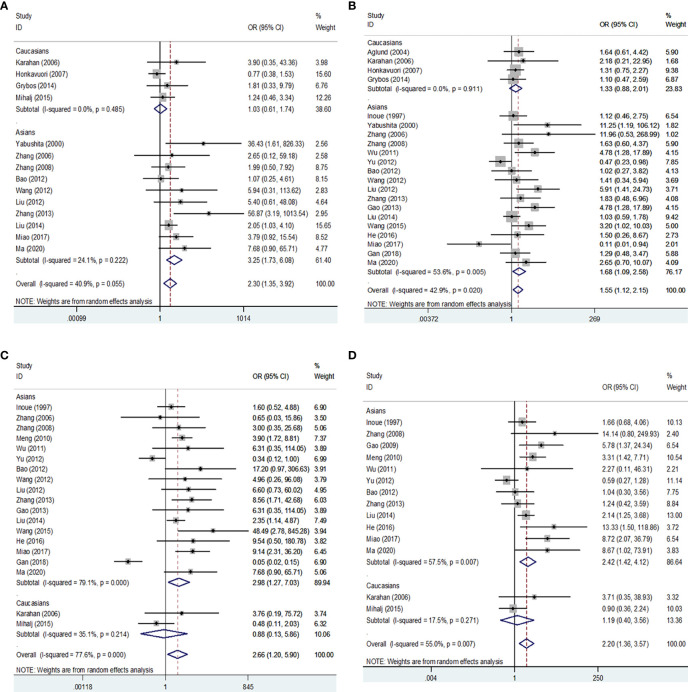
Forest plots for the associations of MMP-9 overexpression with clinical features of EC in Asians and Caucasians. **(A)** Forest plot for FIGO stage of EC. **(B)** Forest plot for endometrial tumor grade. **(C)** Forest plot for lymph node metastasis of EC. **(D)** Forest plot for myometrial invasion of EC. OR, odds ratio; EC, endometrial cancer.

**Figure 4:**
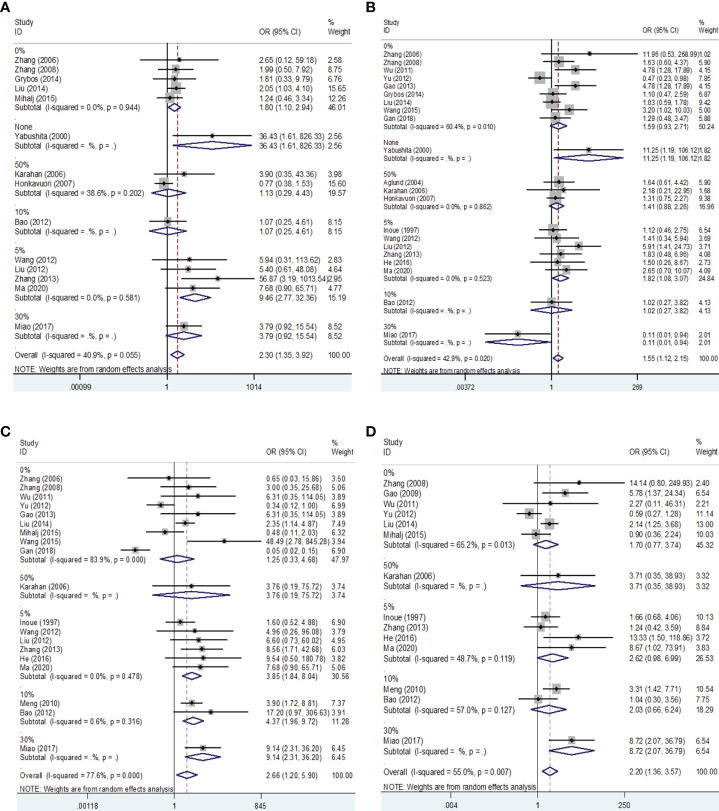
Forest plots for the associations of MMP-9 overexpression with clinical features of EC in different cut-off values of IHC. **(A)** Forest plot for FIGO stage of EC. **(B)** Forest plot for endometrial tumor grade. **(C)** Forest plot for lymph node metastasis of EC. **(D)** Forest plot for myometrial invasion of EC. IHC, immunocytochemistry. OR, odds ratio; EC, endometrial cancer.

Then we performed a meta-analysis to explore the role of MMP-9 overexpression in the clinical characteristics of EC. The results indicated that patients with high G2-G3 grade had higher MMP-9 expression than that with G1 grade in Asians (OR = 1.68, 95% CI = 1.09 – 2.58, *P* < 0.05). And, high MMP-9 expression might represent the III-IV FIGO stage in Asians (OR = 3.25, 95% CI = 1.73 – 6.08, *P* < 0.05). In addition, high MMP-9 expression was significantly associated with lymph node metastasis (OR = 2.98, 95% CI = 1.27 – 7.03, *P* < 0.05), myometrial invasion (OR = 2.42, 95% CI = 1.42 – 4.12, *P* < 0.05) and vascular invasion (OR = 2.67, 95% CI = 1.27 – 5.60, *P* < 0.05) of EC in Asians. Some heterogeneities were found in the analysis of the associations of MMP-9 expression with lymph node metastasis, myometrial invasion and vascular invasion of EC, so the random-effects model was used. Moreover, the subgroup analysis based on the cut-off values significantly reduced the heterogeneity among studies and significant associations were still found (**Figures 3**, **4** and **Tables 2**, **3**). Therefore, the pooled results were convincing. According to the results of the present meta-analysis, some studies have suggested that MMP-9 expression was associated with the development of EC, while others have obtained opposite results which can be seen in **Figure 3**.

**Table 3:** The subgroup analysis based on cut-off values of MMP-9 expression for the risk and clinical features of EC.

Characteristics	Studies	Forest plot analysis		Heterogeneity analysis		Begg’s test
		OR (95% CI)	*P*	*I* ^2^ (%)	*P*	*P*
Risk (Normal vs EC)						
0% (Cut-off value)	8	11.62 (5.28-25.60)	< 0.05	72.200	0.001	0.266
5% (Cut-off value)	6	8.32 (4.91-14.08)	< 0.05	2.300	0.401	0.039
10% (Cut-off value)	2	9.28 (5.27-16.36)	< 0.05	0.000	0.724	–
Tumor grade (G1 vs G2+G3)						
0% (Cut-off value)	9	1.59 (0.93-2.71)	> 0.05	60.400	1.010	0.009
5% (Cut-off value)	6	1.82 (1.08-3.07)	< 0.05	0.000	0.523	0.851
50% (Cut-off value)	3	1.41 (0.88-2.26)	> 0.05	0.000	0.862	
Tumor stage (I vs II+III)						
0% (Cut-off value)	5	1.80 (1.10-2.94)	< 0.05	0.000	0.944	1.000
5% (Cut-off value)	4	9.46 (2.77-32.36)	< 0.05	0.000	0.581	1.000
50% (Cut-off value)	2	1.13 (0.29-4.43)	> 0.05	38.600	0.202	–
Lymph node metastasis (No vs Yes)						
0% (Cut-off value)	9	1.25 (0.33-4.68)	> 0.05	83.900	0.000	0.602
5% (Cut-off value)	6	3.85 (1.84-8.04)	< 0.05	0.000	0.478	0.573
10% (Cut-off value)	2	4.37 (1.96-9.72)	< 0.05	0.600	0.316	–
Myometrial invasion (< 1/2 vs > 1/2)						
0% (Cut-off value)	6	1.70 (0.77-3.74)	> 0.05	65.200	0.013	0.851
5% (Cut-off value)	4	2.62 (0.98-6.99)	> 0.05	48.700	0.119	0.174
10% (Cut-off value)	2	2.03 (0.66-6.24)	> 0.05	57.000	0.127	–
Vascular invasion (No vs Yes)						
0% (Cut-off value)	1	0.43 (0.12-1.49)	> 0.05	–	–	–
5% (Cut-off value)	2	2.67 (1.27-5.60)	< 0.05	0.000	0.617	–
50% (Cut-off value)	1	5.33 (0.27-106.24)	> 0.05	–	–	–
Menopausal status (Premenopause vs Postmenopause)						
0% (Cut-off value)	2	1.02 (0.66-1.55)	> 0.05	35.800	0.212	–
5% (Cut-off value)	1	1.01 (0.25-4.17)	> 0.05	–	–	–
30% (Cut-off value)	1	4.18 (1.11-15.79)	< 0.05	–	–	–

OR, odds ratio; CI, confidence interval; EC, endometrial cancer; cut-off: cut-off values of MMP-9 protein detection with immunohistochemistry; EC, endometrial cancer.

The HR and 95% CI values were extracted from included studies and combined to evaluate the correlation between MMP-9 expression and overall survival of EC. The outcome showed that higher MMP-9 expression represented a worse overall survival of EC (OR = 1.82, 95% CI = 1.01 – 2.62, *P* < 0.05). No significant heterogeneity was found in the meta-analysis for the overall survival of EC (*I* ^2^ = 0, *P* = 0.827). In the three included studies for the overall survival of EC, two studies believed MMP-9 expression affected the survival of EC, while negative result obtained in the study of Yu et al. (**Figure 2** and **Table 2**).

### Publication bias and sensitivity analysis

No significant publication bias was found in the meta-analysis of the present study. In the meantime, the results of sensitivity analysis suggested that the overall results were stable (**Figure 2** and **Tables 2**, **3**).

## Discussion

It has been documented that MMP-9 protein plays a pivotal part in various diseases. For instance, MMP-9 could degrade components of the extracellular matrix and numerous nonmatrix proteins in fibrosis disease. Surprisingly, although MMP-9 levels were increased in the alveolar lavage fluid of idiopathic pulmonary fibrosis patients, MMP-9 promoted abnormal epithelial cell migration and lung tissue repair (45). In the lung fibrosis model of MMP-9^-/-^ mice, deficiency of MMP-9 protected mice from alveolar bronchiolization (46). On the contrary, some studies have shown that MMP-9 overexpression in liver tissue was a risk factor for advanced T category, tumor stage and poor outcome (47, 48). Therefore, MMP-9 might have different roles in diverse diseases. Previous studies have reported overexpression of MMP-9 was associated with the clinical progression of EC (10, 13, 29, 32, 37, 49). However, other studies found no significant associations between MMP-9 overexpression and the clinical stage of EC (50, 51). Furthermore, many inconsistent results involving the associations between MMP-9 expression and clinical features of EC were reported (10–37). Therefore, the role of MMP-9 overexpression in EC needs to be studied urgently. In fact, functional studies have showed that MMP-9 was expressed in proliferative phase endometrium, hyperplastic endometrium and EC (52, 53). In the peritoneal endometriotic lesions, positive cells (59.1%) were more than colorectal endometriosis (44.4%). Nevertheless, EC patients had the highest levels of MMP-9 expression (54). Therefore, the expression level of MMP-9 might increase with the development of endometrial disease.

This was the first meta-analysis assessing the associations of MMP-9 overexpression with EC risk, clinicopathological features and prognosis. The results indicated the significant associations between MMP-9 expression and EC risk. However, only one included study was performed on Caucasians for the analysis of risk. So, the overall results for the risk of EC might be more applicable to Asian population. Furthermore, stratified analysis based on the cut-off values of MMP-9 expression significantly decreased the heterogeneity among studies and a significant correlation for risk was still found in the stratified analysis, which showed that the results of the meta-analysis were convinced. It’s worth noting that MMP-9 expressed highly in EC patients with G2-G3 grade, III-IV FIGO stage, lymph node metastasis, myometrial invasion, vascular invasion or postmenopausal, indicating that high MMP-9 expression promoted clinical progress of EC. According to our results, ethnicity might be an important influencing factor because MMP-9 overexpression mainly promoted the clinical progression of EC in Asians. One meta-analysis has also found MMP-9 expression of bladder cancer tissue presented significant race diversity (55). Therefore, MMP-9 expression might have a potential association with ethnicity. In the meantime, stratified analysis based on the cut-off values of MMP-9 expression also significantly reduced the heterogeneity among studies for clinical features of EC, which hinted different cut-off values of MMP-9 expression were used in the included studies of the meta-analysis. Besides, the overexpression of MMP-9 was significantly associated with a worse prognosis for EC, which demonstrated MMP-9 overexpression reduced the survival time of EC patients. Therefore, the expression level of MMP-9 should be reduced to improve the prognosis in the clinical treatment of EC patients. Moreover, no significant heterogeneity among included studies for the association of EC survival with MMP-9 overexpression was found, which indicated that the pooled result was convincing. Sensitivity analysis revealed that no individual study significantly affected the overall results which showed that the pooled results were stable.

Several limitations of the present study should be pointed out. First, the number of included studies conducted on Caucasians was too small for the meta-analysis of EC risk. Second, cut-off values of MMP-9 expression were evaluated dependent on included studies for the associations of EC clinical features with MMP-9 overexpression, which significantly affected the heterogeneity among the eligible studies. Third, other factors might be involved in the heterogeneity among the included studies. However, these factors were not quantified or provided in the eligible studies. Thus, more multi-center studies with more clinical information were warranted to verify the role of MMP-9 overexpression in EC.

## Data availability statement

The original contributions presented in the study are included in the article/**Supplementary Material**. Further inquiries can be directed to the corresponding author.

## Author contributions

All authors listed have made a substantial, direct, and intellectual contribution to the work, and approved it for publication.

## Conflict of interest

The authors declare that the research was conducted in the absence of any commercial or financial relationships that could be construed as a potential conflict of interest.

## Publisher’s note

All claims expressed in this article are solely those of the authors and do not necessarily represent those of their affiliated organizations, or those of the publisher, the editors and the reviewers. Any product that may be evaluated in this article, or claim that may be made by its manufacturer, is not guaranteed or endorsed by the publisher.
